# Eight Hours a Day

**Published:** 1869-07

**Authors:** 


### HALL'S JOURNAL OF HEALTH.

VOL. XVI. JULY, 1869. No. 7.

### EIGHT HOURS A DAY

Entitles a government worker to a full days' wages, according to a law recently enacted by Congress, and many members of our State Legislatures have favored the passage of the Congressional law. It seems to us that no man, not absolutely bereft of reason could sincerely advocate such an opinion; but as men do believe that such a law is expedient and just, who have a thousand times more sense than we have, it comes within the range of possibilities that we are a more fit candidate for an insane asylum than the legislators who vote to obtain votes. Some one has made the calculation, that if every body worked four hours out of the twenty-four, every body would have a comfortable living. But no practical result is likely to follow such an item of information, because every body won't work four or any other number of hours every day. From the year one to the present time the world has been of two classes, the riders and the ridden; and as long as the world stands, or wheels, those who work will have to support those who won't work, unless the Millennium changes the face of things. Meanwhile let us look the facts straight in the face, admitting that those who have thought differently were sincere in their belief, that a man should be paid a full day's wages for eight hours' work.

If I want to build a house to rent out, and the men who build it charge as much for eight hours work as they would for twelve hours work, then the house costs fifteen thousand dollars instead of ten, hence it must rent at a rate which will give me legal interest. Thus, the more it costs to build a house, the more the mechanic who rents it has to pay; and when the mechanic goes to purchase meats and groceries, he must pay just that much more for these articles, as the men who sell them to him have to pay proportionately higher rent, and must charge proportionately higher prices; so wherever the mechanic goes with his day's wages, he must pay in proportion, whether for clothing, or food, or house rent, and the result is, that the money he receives for an eight-hour day's wages, goes no farther than the old-fashioned day's wages. Thus far they are even. But look at the many hours of good weather lost in a week. Eight hours are from eight in the morning until four in the afternoon; and if it is a good out-door working day, there are four hours during which he is earning nothing; the next day is perhaps a rainy day and he can earn nothing, hence that whole day is lost, and four hours of the preceding day; had he worked those four hours, half a day's wages would have been saved to his family. I look out upon Mr. Stewart's grand enterprise for giving honest, industrious women of good character, elegant rooms and good board for two or three dollars a week, so that they may save all they earn over that, for future use; to repeat, I look out upon the noble work every summer's morning from my chamber wiudow, at four-and-a-half o'clock, the air is so pure and cool, but I see no workmen there; at five, none; at six, none; where can they be! What a pity to lose all these lovely hours for work! At seven o'clock they begin a days' work. Another morning I look out and it is raining; hour after hour it rains; and there I see the men in little groups, some under a shed, others under a door stoop, or friendly awning; when I pass them as I go down to my office on such occasions, they look so sad; because they are to live to-morrow, on what they earn to-day; but they are earning nothing and their rent is going on, and the wife and children at home are getting hungry, and they are sad too, for they know "father" is earning nothing, and he will come home wet and hungry and sad as they are; and yet, on yesterday, the beautiful hours in the cool of the morning, when they could have worked to such advantage, they were doing nothing. It is pitiful to think of.

If your tenant wants some thing done, and who ever had a tenant that did not want something done, every time he comes to pay the "rent," as if he considered that much at least saved, or as if he took a malicious satisfaction in making his landlord disgorge as much as possible for repairs, you send for your painter or carpenter or plumber; they all charge you at the rate of—not eight hours a day, but about two hours; well, the landlord cares very little about it, for it is added to the rent of next month or next year, and, yet renters are the most unhappy people in the world; to credit their story, they are perfect martyrs to high rents; they assure you that every cent they can rake and scrape they have to pay to the landlord. My dear day-laborer, capital is king; always has been king, and always will be until Congress passes a law that nobody ought to work, and that other people ought to support them. Come to think it over, it would be a grand plan, it would be getting at the root of the matter in double-quick time; only go a mite deeper, and pass a law that every body should have a beautiful little cottage made over to him, with three or four servants and a liberal annuity; thus, abolish all work and "nobody will have nothin' to do, and plenty of time to do it in." These will be the merry days of Uncle Samuel, and future ages will sing of them with "solemcholy rapture."

It has been argued in Congress, the man who made the argument stated that he spoke from actual experience, that he could not work to advantage longer than eight hours a day; that he had observed while working by the day in his own person, that he could do as much in eight hours as in twelve He may have done so while working for another or by the day, but we do not believe that there are two men in a million who, working at eight hours a day, will accomplish as much as if twelve hours had been spent in work. We have to do with men as they are, and not as they ought to be.

Girard, and George Peabody, and Peter Cooper, and A. T. Stewart, and Cornell, and other grand names, immortalized by princely beneficences, never made their fortunes by working only eight hours a day; many a time and oft have they worked day and night, making the average of a life-time not eight, but from twelve to fifteen hours.

The man who has to depend on his own exertions for a living, and who is satisfied to work only eight hours a day, is no man at all; he is a born loafer, an innate idler, and never will be worth a button unless he steals it or cheats somebody. The man of thrift cannot be hired to waste half an hour of daylight; he would consider it lost money; he would fairly groan under the necessity of such a waste of time.

The man who loves work as a means of bettering his condition, and who has an ambition to lay up something for a future day, never finds a day too long; he begrudges the early sun-down; and leaps from his bed at the peep of day. But mind, we do not say he ought to do such a thing. We merely speak of the promptings of a noble and ambitious spirit.

Much has been said about the necessity of giving a man a chance to improve himself. That is very beautiful in theory; nine-tenths of the clerks in our stores, with a fixed monthly salary, would be very glad to have the salary and strike off at three o'clock in the afternoon, not only on Saturdays, but every day, and have the remaining hours to improve themselves in walking up Broadway; in riding in Fifth Avenue stages, and putting their feet under ladies' dresses, and pushing against their feet, as we have known tbem to do. We don't see the wisdom or the morality of allowing a young man or anybody else to improve his time at the expense of the man for whom he is working; any man who wants to do it under the subterfuge of an eight hour law will never be worth a copper of his own laying up.

If day-workers want to improve their minds, let them do it on rainy days, when they can not work out of doors; and during the long winter nights. Intelligent employers in every department of business in New York know very well that since the wages of workmen have been increased, and their time of work diminished, the workmen have become more saucy; drink more whisky; have less money, and are less faithful than they previously were, and these vices are increasing. Yet these are the very men who are so constantly complaining of the high prices of every thing, as a reason for demanding higher wages; if mechanics demand higher wages from those who employ them, they must also pay higher prices to those whom they employ to serve them with food and clothing and shelter. We must all see that as soon as it costs less to build a house, the landlord will rent it at a less price, and the butcher can sell his meat at a less profit, and the merchant his goods, because they have to pay less rent. Demanding the same price for a less number of hours work, is the same as charging more for the old time days-work. The fact is, the mechanic wants more for his work than it is worth; he wants to make a man pay more money for his labor than the man wants to pay, in other words, democracy is becoming a despotism and universal suffrage an ineffable stupidity, under the circumstances of the present age. All "strikes" are despotisms; their inevitable tendency is to free trade. If my tailor charges me fifty dollars for making me a coat, then people who want coats will send to some other country for coats which can be made quite as good for twenty dollars; and then the poor man will see that twenty dollars would get him as good a coat as fifty, if he did not have to pay government the extra thirty dollars; to save him that thirty hard-earned dollars he will vote for free trade, which will give us every thing cheaper; what we use will be made abroad, and we will have no use for eight-hour men, or any other kind of workmen. The eight-hour law violates the fundamental principle that we should do to others as we would have others do to us; we take the meaning to be this in reality, that a man should be as faithful to his employer as he would be to himself. If a man is working for himself as a joiner or shoemaker or tailor, will he stop work at the end of eight hours? and yet he wants me to pay him as much for eight hours work as if he had worked all day.

The mass of workers in this country are democratic; they boast of a free country, where a man can do as he pleases, so long as he does not interfere with his neighbors' rights; and yet every man who "strikes" for higher wages, is endeavoring to compel his neighbor to pay him more for his work, than he wants to pay; if his neighbor will not pay, he will not only not work for him, but he will break the head of any other man who would do the work, and whose wife and children are that moment starving for the food which his work would pay for. Plenty of work to be done, and nobody allowed to do it. Radical democracy in the United States is, that every man shall do as he pleases, aud make every body else do exactly as suits him; making it here, as we have already stated, that in this country democracy is a despotism; not an intelligent despotism; it is the despotism of ignorance and selfishness. Better let us go back to the olden time when honest men worked from sunrise to sunset, at stipulated wages; if the wages could not be agreed upon, then the worker could go and find some one who would give him what he asked, and the employer would engage a man who was willing to take what he offered. There was no compulsion on either side, and all parties were free.

A man can not do as much work in eight hours as in twelve, yet he and his family must eat the same amount of food, use the same amount of clothing and pay the same rent, so that at the end of the year he will be out of pocket by one-third; that is, when he does his own work on the principle of eight hours a day; and to a certain extent it is the same thing if he works only eight hours for another. Every thing seems to be going on swimmingly just at this time; men are getting nearly twice as much for work as they used to. But they will all tell you that they cannot lay by as much as they used to; and it is an undeniable fact, that the rich are getting richer, and the poor poorer. It is, in part at least, because men do not work as much every day as they ought to do; and the lost time has to be accounted for at the end of every year; that is to say, working men can not earn a living for themselves and families by working only eight hours a day. A man can not produce in eight hours as much as he will consume in twenty-four, under the present organization of society; and every attempt "to get along" with that amount of daily labor, will eventually fail.

We have been speaking mainly of out-door workers, in order to make the matter plainer; but the same principles apply to those who work in-doors by the day, or by the job or piece; because in all cases, the principle is equally involved, of wanting more for a thing than it is worth, which is unjust the world over; and injustice will always, sooner or later, bring upon itself its merited punishment.

The experience of five thousand years is, that individuals and communities must be kept employed, if they would be kept out of mischief. Hooray! for that immortal little fellow, who one day went to see a friend, or rather two friends, a gentleman and his wife; after a little chat, they asked him not to be in a hurry; and he remained one night; then began to be ashamed of sponging on the hospitality of the house; but when he proposed leaving they requested him not to be in a hurry, and he found it so delightful; he was made so welcome; made to feel so much at home, that he forgot all about being from home; and staid and staid, and wrote poetry for seventeen years. One of the verses was this:

"In works of labor or of skill,
I would be busy too,
For Satan finds some mischief still,
For idle hands to do."

But Isaac Watts only expressed in poetry what Joseph put in practice when he became ruler in Egypt. He kept the people busy, he gave them work, and not only so, he scattered them, kept them apart so as not only to keep them busy, but kept them from hatching mischief. It is pretty safe to say that the French Government would not stand a month, if the workmen of Paris were not employed; nor would England be saved from anarchy if the working people were not kept busy earning their daily bread; no government on earth can stand if the masses are not kept employed; nor will the United States be an exception to the general rule under the present state of things. Let every man work but eight hours a day, and there are eight other hours for idling, for huddling together and concocting wicked schemes; for drinking and smoking and gambling and the indulgence of obscene conversation; of sentiments contrary to good morals and right government. A few persons, one in ten thousand might employ the time in excess of the eight hours in "improving the mind," but the great masses would loiter about corner groceries or drinking saloons, and other demoralizing places, and by having more leisure to drink and smoke and gamble and attend theaters, Negro minstrels and circus performances, would inevitably grow more and more demoralized, and idle and dissatisfied and worthless. The laboring classes, the poor, are the worst enemies of one another, not designedly it may be, for the poor are generally good-hearted, but inadvertently, because they grievously oppress one another, and thus aggravate the hardness of each other's lot. See how many of the small tradespeople in New York do this all the time, by selling watered milk, giving short weight at butchers' stalls, and scant measure at the corner groceries. A rich man pays six dollars a ton for his coal; the poor sewing woman who gets it by the pailful, pays twelve. In the matter of mechanics' wages, the same inequalities are in operation all the time; thus, it costs as much to feed and clothe and shelter the family of a horse-car conductor as that of a stone-mason or bricklayer; but these latter demand five dollars for eight hours work, while the former will work very cheerfully sixteen hours out of the twenty-four, for two dollars and a half; and yet the mason makes the car conductor pay the same prices for meat and bread that he does himself, when the car conductor works twice as long for half the money; really getting four dollars for work for which the conductor gets one. The mason does this by making it cost so much to build a house, in consequence of his working so short a time for so large an amount of money; and house-rent in cities regulates the prices of all that a family consumes.

This is the way in which a doctor-editor looks at "strikes," and the eight hour law; but as mechanics and laboring men are, after all, the great supporters of society, and by their industry as a class, and their law-abiding principles, and their honesty of purpose, and their enduring patience under their lives of toil, merit the respect of all good men, we propose a return to the good old times of the past, by taking a compromising backstep as a beginning; and let all this tom-foolery of allowing the laboring classes "more time for improvement," go to grass. As a class, they either don't want improvement, or can't spare the time; they want more money more than more improvement. Four hours a day, "a Congressional day!" O you fellows at Washington, who sold your principles for votes last winter, in making a law, so expressed, that the rich man could interpret it in one way and a poor man the reverse, so that you might gather in all their votes and thus secure yourselves for another term; yes, passed a law so loosely expressed, that it meant everything and nothing at the same time; and not even a Philadelphia lawyer could tell which was which, and at last decided that if it did not mean this, it did mean that, or 'tother, or nothing; and being really afraid to commit himself, referred it to the Soldier who feared nothing, and Grant-ed the poor man's request, just for the sake of letting him see, a little later, that he wanted a very unwise thing; but we have wandered from the track, as other resistless locomotives sometimes do; but our plan will be all the more believed one of these days, and will show how much good sense some editors have; how they coin great practical truths from their brains in their cozy upper-rooms on great, busy, bustling, whirling Broadway. Our plan, if carried out, will give the mechanic more money at the end of every day, and the man who employs him will be better satisfied. Don't be in a hurry, reader. You are "in for it," for a life of toil, but we want you to have more money, so that you may be able to give your children more schooling and more church; that by being able to start in the world with more information and with better opportunities of moral and religious training than you had, they may work to better advantage and for higher pay than you; work twelve hours for six dollars, instead of eight hours for five dollars and in the same proportion as to all other rates of progress. Thus, the builder erects a tenement for less money, will be able to rent it for less, and in time, every thing else will cost less; at the same time, you will be earning more money every twenty-four hours than you now do, and you will be wasting no time, as you now are doing. This plan does not apply to those who work by the job; but let them work longer and charge less for it. The present state of wages can not stand; in the very nature of the case, the working man must go to the wall, will come out second-best, because capital is king.

